# Melioidosis after a long silence in Sri Lanka: an environmental hazard and dilemma in diagnosis, with recovery and longitudinal follow-up for 13 years: a case report

**DOI:** 10.1186/s13256-020-02372-2

**Published:** 2020-04-18

**Authors:** Manoji M. K. Pathirage, Senanayake A. M. Kularatne, Kosala G. Weerakoon

**Affiliations:** 1 Department of Medicine, Faculty of Medicine, University of Peradeniya, Peradeniya, Sri Lanka; 2 Department of Parasitology, Faculty of Medicine and Allied Sciences, Rajarata University of Sri Lanka, Mihintale, Sri Lanka

**Keywords:** *Burkholderia pseudomallei*, Melioidosis, Sri Lanka

## Abstract

**Background:**

Melioidosis is a potentially fatal bacterial infection caused by *Burkholderia pseudomallei*. The existence of melioidosis in Sri Lanka was once unheard of, and entertaining it as a diagnosis in clinical practice was extremely rare.

**Case presentation:**

In this case report, we describe the clinical, epidemiological, and longitudinal follow-up data of a 58-year-old previously healthy Sinhalese woman who presented to our hospital with protracted febrile illness of 5 weeks’ duration, later developing multiple abscesses at different sites of the body. There was a significant delay in confirming the diagnosis of melioidosis by isolating *B. pseudomallei* from blood and pus cultures. The patient recovered fully with a prolonged course of antibiotics and has remained in good health over the last 13 years without recurrence. Despite being immunocompetent, she had contracted the infection by a brief contact with mud soil in a footpath.

**Conclusions:**

A high index of clinical suspicion along with laboratory support is needed to confirm the diagnosis of melioidosis. Treatment with sensitive antibiotics over a long duration is needed, and longitudinal follow-up is essential to detect recurrences. This case raised awareness and created renewed interest in studies of melioidosis in Sri Lanka.

## Background

Melioidosis is a potentially fatal infection caused by a gram-negative, aerobic, non-spore-forming, motile bacillus, *Burkholderia pseudomallei*, that is found in soil and water. It was first described in 1912 by a pathologist, Captain Alfred Whitmore, and his assistant, C. S. Krishnaswami [1, 2]. Melioidosis is endemic in South and Southeast Asia, particularly in Thailand and Northern Australia [1–3]. Even though Sri Lanka is situated in the endemic belt of melioidosis, the disease had been considered uncommon on the island, probably due to underreporting and lack of awareness. However, the first case of melioidosis from Sri Lanka was diagnosed in 1927 in a European tea broker, being the third country to report melioidosis in the literature [4]. The clinical spectrum of the disease may range from an acute fulminant septicemia (e.g., pneumonia, arthritis) to a chronic infection characterized by multiple abscesses forming at different sites in the body. In this article, we report the clinical details of a severe case of melioidosis in Sri Lanka that posed a diagnostic dilemma and in which the patient made a full recovery from the infection and has remained in good health for the last 13 years without recurrence.

## Case presentation

In February 2006, a previously healthy 58-year-old Sinhalese woman presented to our hospital with a history of intermittent fever, headache, loss of appetite, loss of weight, dry cough, and arthralgia of 5 weeks’ duration. She had no known past medical conditions or any family history of chronic disorders or similar illnesses. She was a housewife who frequently engaged in gardening and had no history of visits to forest areas or working in paddy fields. She was not alcoholic and was a nonsmoker.

At the beginning, she had been hospitalized, had undergone investigations, and had received broad-spectrum antibiotics. Due to the nature of the protracted illness with fever, and because she had an erythrocyte sedimentation rate (ESR) above 100 mm/h and increased C-reactive protein (CRP), a course of antituberculous antibiotics had been given over 2 weeks without definitive confirmation of the diagnosis of tuberculosis. While on antituberculous medications, instead of clinical improvement, she showed further deterioration of her illness and developed new problems, including appearance of a tender lump over the right temporal area over the course of 1 week and another similar lump over the anterior aspect of the left thigh of 5 days’ duration. At this juncture, she left the first hospital at her own wish and secured admission to Teaching Hospital Peradeniya (THP).

On admission to THP, she looked very ill and feeble and was febrile and tachypneic. She had a tender lump resembling an abscess over the right temporal area. Furthermore, she had similar lumps over the anterior aspect of the left thigh. She had an inflamed, swollen knee and wrist joints with a tender erythematous rash over both shins that resembled erythema nodosum. Her pulse rate was 110 beats/min with blood pressure of 100/65 mmHg. The results of her respiratory system and abdominal examinations were clinically normal. She was conscious, rational, and had no neck stiffness, with a clinically normal neurological examination result.

Blood samples were taken for basic investigations and culture at this juncture. Diagnostic needle aspirates from the cystic lumps and knee joint effusions were purulent and were sent for bacteriological investigations. Blood samples for cultures were taken under aseptic conditions. Three separate blood cultures were taken in 30-minutes intervals and drawn into standard blood culture bottles. Knee joint and skin lump aspirates also were drawn into separate culture bottles under aseptic conditions. All the samples were transported to the microbiology laboratory, which is located within the hospital premises, and the cultures were done by a consultant microbiologist. Pending investigations, the patient was treated with intravenous (IV) co-amoxiclav (amoxicillin mixed with clavulanic acid).

On the fifth day of the patient’s admission, she became drowsy and developed focal seizures involving the right upper and lower limbs and lasting for around 2 minutes. She became drowsy with a Glasgow Coma Scale (GCS) score of 12/15. She moved all her limbs, and her deep tendon reflexes were normal, but she had an upgoing plantar response. She had no neck stiffness, positive Kernig sign, or focal neurological signs. She had persistent tachycardia with low blood pressure of 90/50 mmHg. Her respiratory rate was 30 breaths/min, and she had bibasal crepitations. She had bilateral knee joint effusions, and her knee joints were swollen, warm, erythematous, and tender. A tentative diagnosis of cerebral abscesses was made, and the patient was transferred to the intensive care unit.

Urgent contrast-enhanced computed tomography of the brain showed a well-defined cystic mass in the right temporal region outside the skull vault and contrast-enhanced changes in the adjacent brain matter. Furthermore, a 12-lead electroencephalogram (EEG) showed a theta-wave focus over the right temporal area with intermittent epileptiform discharges compatible with structural brain damage. The results of her ultrasound examination of the abdomen and an echocardiogram were normal. Her white blood cell count was 3.3 × 10^9^/L with neutrophils of 83% and lymphocytes of 12%. Her ESR was 120 mm/first hour, and her CRP level was 192 mg/L. Her chest x-ray showed multiple peripherally located, ill-defined cystic areas in both lung fields, more in the lung bases, suggestive of multiple lung abscesses. By that time, the blood culture, knee joint aspirate, and skin lump aspirates grew *B. pseudomallei*, which was confirmed by the local microbiologists with the help of international reference laboratories [5]. Thus, a diagnosis of melioidosis was confirmed, and treatment commenced.

Following the confirmed diagnosis her antibiotic regimen was changed and started on IV meropenem 1 g 8-hourly for 8 days and later changed to IV imipenem 500 mg 6-hourly, based on the antibiotic sensitivity pattern of the bacterial isolate. Two more antibiotics were added to the regimen at the same time: IV ciprofloxacin 400 mg 12-hourly and IV ceftazidime 2 g 6-hourly. The patient showed gradual improvement of her general condition, and her fever subsided in a few days. However, all three antibiotics were continued for 30 days and changed to an oral antibiotic course comprising cotrimoxazole 1920 mg twice daily plus doxycycline 100 mg twice daily for 20 more weeks. Her seizures were controlled with sodium valproate 200 mg thrice daily. In follow-up, the patient’s chest x-ray, ESR (50 mm/h), CRP (0.7 mg/dl), and liver and renal profiles showed gradual improvement, and the results of her retroviral screening were negative. Her blood sugar and hemoglobin A1c levels were within normal limits. The result of repeat EEG in 3 months’ time was normal, and the patient’s sodium valproate was gradually tapered. After 6 months of antibiotic treatment, her ESR was 12 mm/first hour; her CRP was < 6 mg/L; and all of her other biochemical parameters were normal. Then, she was advised to come back to our hospital for annual screening to check for recurrence of the disease, including ESR and CRP measurement. She has had no recurrence of her illness during the last 13 years and remains well.

Subsequently, how she contracted the infection was inquired about. She was able to recall contact with soil a few days prior to developing her illness. The footpath leading to her home had a side drain that was filled with mud and soil. She found that a worker had dug up the drain and piled mud and soil on the footpath. The patient, dismantled the piled-up soil with her bare feet and made the footpath accessible.

## Discussion and conclusions

This case reveals the existence of melioidosis in Sri Lanka and opened a floodgate to research and detection of more and more cases. The case highlights the nature of the infection as a great mimicker of tuberculosis. Also, our patient was a healthy, immunocompetent individual who still enjoys a healthy life, even more than a decade after her full recovery. This is contrary to the knowledge of the risks when contracting melioidosis. The typical epidemiology of contact with mud and the saprophytic nature of the pathogen are reiterated in a new tropical geographical area. The description of the intense nature of treatment and follow-up will help in case management and provide a lesson for posterity.

Our patient is an immunocompetent healthy adult who contracted melioidosis from direct skin contact with muddy soil in a footpath. Despite delay in diagnosis and development of life-threatening complications, she made a full recovery after receiving a prolonged course of antibiotics. Fortunately, she has not experienced any relapses over the last 13 years and remains healthy. Her illness began as a prolonged febrile illness with many parameters similar to tuberculosis. Late development of septic arthritis, multiple subcutaneous and lung abscesses, and even involvement of the brain pushed her into life-threatening condition. However, identification of *B. pseudomallei* from cultures helped us to prescribe appropriate antibiotics despite having lost a considerable amount of time. Because she was in good health and without comorbidities, she was able to overcome the disease. This case set a precedent to entertain melioidosis in the differential diagnosis of obscure septic illnesses and led to detection of more and more sporadic cases in Sri Lanka.

The term “melioidosis” derives from the Greek *melis*, distemper of asses, *oeidēs*, resemblance, and *osis*, a suffix indicating an abnormal condition or disease. It can present with a broad spectrum of clinical signs and symptoms and may be latent for months or years before the initial clinical manifestations. It is well known that it particularly mimics tuberculosis. Clinical melioidosis has a diverse spectrum of presentations, ranging from localized infection to acute pneumonia and fulminant septic melioidosis. Our patient’s case qualifies for all of the above known signs and symptoms described in the literature.

Melioidosis is predominantly a disease of tropical climate, and the pathogen is an environmental saprophyte [6]. The incidence of the disease is high in Thailand (2000–3000 clinical cases annually) [7] and is becoming an emerging infection in India [8]. It is a disease of the rainy season in endemic areas. Occupational exposure is the major risk factor for the disease. Severe infection is an opportunistic disease and more commonly seen in patients with diabetes, chronic renal disease, chronic lung disease, cirrhosis, thalassemia, alcoholism, and other conditions that cause immunosuppression [9].

Despite being a healthy adult, our patient contracted the infection from casual contact with soil. Probably, the muddy soil in the drain was rich with the pathogen. Information of this nature would help improve knowledge of the epidemiology of the disease in Sri Lanka and to plan prevention and control strategies [10].

Since this index case, melioidosis has been considered as an emerging infection in Sri Lanka and has created renewed interest among clinicians and microbiologists, leading to an increased case detection rate [11, 12]. However, we assume that lack of awareness of the disease leading to underdetection of cases and underreporting has underestimated the true burden of the disease. Hence, a high index of clinical suspicion is essential to making an early diagnosis, because this is a treatable condition. The current awareness about the disease led to reporting of ten confirmed cases of melioidosis in Sri Lanka in 2013, with one fatality. Since then, around ten cases have been reported addressing the complications related to melioidosis, such as sacroiliitis, pneumonia, and endocarditis [13–16].

The diagnosis of acute or chronic melioidosis remains challenging because strong laboratory backup with bacteriology is needed. Establishment of laboratory facilities is very essential because isolation and identification of organisms from various clinical specimens remains the gold standard of definitive diagnosis. Positive bacterial culture and identification take several days, leading to delay in the initiation of appropriate therapy for melioidosis. Undue delays in commencing treatment and the presence of comorbidities lead to a high fatality rate in the disease. Being a saprophyte, *B. pseudomallei* is generally resistant to many antibiotics. However, there are antibiotics that are effective against it, including tetracycline, trimethoprim-sulfamethoxazole, chloramphenicol, third-generation cephalosporins, amoxicillin-clavulanic acid, ureidopenicillins, carbapenem, cefoperazone/sulbactam, and azithromycin [17, 18]. Despite long delay in confirming the diagnosis, our patient responded to high-dose IV meropenem followed by imipenem. A further long course of oral antibiotics helped to achieve complete cure of her infection because she remained well without recurrence. Even though the cumulative cost of antibiotics was very high, it was not a burden for the patient because of the availability of free medical care in Sri Lanka.

In conclusion, we report a case of melioidosis in Sri Lanka, highlighting the environmental impact on human health, challenges in diagnosis, some aspects of epidemiology, and long-term follow-up. A high index of clinical suspicion along with laboratory support is needed to confirm the diagnosis. Treatment with sensitive antibiotics over a long duration is needed, and longitudinal follow-up is essential to detect recurrences. Our patient is extremely lucky to have recovered from this dreaded infection and to have remained free of recurrences. We hope that sharing this case history with readers will expand their knowledge and renew interest in melioidosis in Sri Lanka.

## Data Availability

Not applicable.
